# Editorial: Antimicrobial resistance in aquatic environments, volume II

**DOI:** 10.3389/fmicb.2023.1211464

**Published:** 2023-05-11

**Authors:** Jorge Olivares-Pacheco, Elisabet Marti, Lorena Rodríguez-Rubio, William Calero-Cáceres

**Affiliations:** ^1^Grupo de Resistencia Antimicrobiana en Bacterias Patógenas y Ambientales GRABPA, Instituto de Biología, Facultad de Ciencias, Pontificia Universidad Católica de Valparaíso, Valparaiso, Chile; ^2^Millennium Initiative for Collaborative Research on Bacterial Resistance (MICROB-R), Santiago, Chile; ^3^Department of Food Microbial Systems, Agroscope, Bern, Switzerland; ^4^Departament de Genètica, Microbiologia i Estadística, Universitat de Barcelona, Barcelona, Spain; ^5^UTA-RAM-One Health, Department of Food and Biotechnology Science and Engineering, Universidad Técnica de Ambato, Ambato, Ecuador

**Keywords:** antimicrobial resistance (AMR), aquatic environment, One Health, sewage, rivers/streams, ARGs antibiotic resistance genes, genomics

An integrated approach that encompasses all ecological compartments is urgently needed to understand the emergence, maintenance, and dissemination of multidrug-resistant bacteria and antimicrobial resistance genes (ARGs) (Hernando-Amado et al., 2019). However, the role of aquatic environments in this process remains an open question for the scientific community. In light of this, we collaborated with Frontiers in Microbiology to launch a Research Topic “*Antimicrobial resistance in aquatic environments*” during 2020–2021. This topic aimed to deliver state-of-the-art knowledge and ideas on the role of aquatic environments in selecting, maintaining, and dispersing antimicrobial resistance (AMR) determinants. It included articles from Europe, Asia, America, and Africa that expanded our knowledge and raised new questions for the global scientific community (Calero-Cáceres et al., 2022). Due to its success, we launched a second volume of the Research Topic in May 2022.

This volume received 8 manuscript submissions, all of which underwent strict peer review. Of these, 5 were accepted for publication. The Research Topics covered a range of subjects, including the evaluation of anthropogenic pollution in water bodies, bioinformatic re-analysis of whole-genome sequences of *Escherichia coli* harboring mobile colistin resistance, a review article on cross-selection of antibiotic resistance in aquatic environments, and a meta-analysis of the prevalence of antibiotic-resistant bacteria (ARB) in marine bivalves. These studies employed modern methodologies such as high-throughput qPCR and whole-genome sequencing.

## Evaluating anthropogenic impact on water bodies: antibiotic resistance determinants and biological tracers of pollution

Two articles in this volume evaluated the anthropogenic impact on water bodies in terms of antibiotic resistance determinants and biological tracers of pollution. The first study, conducted by Fernanda et al. in China, assessed the diversity and abundance of ARGs and mobile genetic elements (MGEs) in Taihu Lake's inflow rivers. Results showed a significant correlation between water quality parameters and ARG abundance, as well as a strong positive correlation between ARGs and industrial, low-density residential, and cultivated land. These findings indicate that Taihu Lake's inflow rivers are polluted by multiple sources, including nutrients and high ARGs abundance.

The second study was conducted by Nguyen et al. at two sites along the Chattahoochee River in the United States to understand how within-day variation could impact monitoring data interpretation. Samples were collected and assayed for fecal indicator bacteria (FIB), human fecal-associated microbial source tracking (MST) markers, and clinically relevant ARGs. Mean levels of FIB and ARGs were similar across sites, while MST markers occurred at higher mean levels at the natural site. Human-associated MST markers positively correlated with ARGs at both sites. In addition to their relevance in their specific geographical areas, these results provide valuable insights for potentially standardizing tracers for ARGs dissemination in the environment.

## The importance of One Health in addressing multidrug-resistant bacteria

In their article, Calero-Cáceres et al. discussed the importance of the One Health concept in addressing multidrug-resistant bacteria. They presented an analysis of whole-genome sequences of *E. coli* that harbor the mobile colistin resistance gene *mcr-1* from various origins and geographical locations. The results revealed a wide array of ARGs, virulence genes, and plasmids in *E. coli* isolates that carry *mcr-1* genes worldwide. These findings underscore the importance of analyzing environmental settings as part of surveillance programs to understand the origins and dissemination of *mcr* genes.

## Prevalence of antimicrobial resistance in bacterial isolates from bivalves

This study of Albini et al. aimed to describe the prevalence of AMR in bacterial isolates from bivalves using a systematic review of the literature. The meta-analysis revealed *Aeromonas* spp. as the genus with the highest prevalence of AMR (37%), followed by *Vibrio* spp. (34%), *Salmonella* spp. (18%), and *E. coli* (15%). Resistance to third/fourth/fifth generation cephalosporins and fluoroquinolones was recorded in ~10% of *E. coli* isolates. The study shows the presence of ARB, including bacteria resistant to the highest priority critically important antimicrobials in marine bivalves, posing a risk for consumers.

## The impact of increased disinfectant and antiseptic use on wastewater and water bodies

The review of Basiry et al. addressed the issue of increased use of disinfectants and antiseptics (DAs) due to the outbreak of the SARS-CoV-2 pandemic, resulting in higher concentrations of these compounds in wastewaters and water bodies. Their constant presence in water bodies may lead to development and acquisition of resistance against the DAs and may also promote antibiotic resistance (AR) due to cross- and co-selection of AR among bacteria that are exposed to the DAs. The review provides an overview of DAs structure and their mode of action against microorganisms. Relevant examples of treatment techniques to increase the DAs removal efficiency from wastewater are discussed. Research needs to determine the impact of DAs in wastewater treatment plants and the consequences of their presence to the spread of AR.

In conclusion, these studies provide an excellent update on the role of aquatic ecosystems in the evolution and dissemination of AMR globally. The editors encourage the scientific community to take note of the results and challenges presented in this Research Topic.

## Author contributions

JO-P, EM, LR-R, and WC-C contributed to analyzing the reviewed articles and preparing the manuscript. All authors contributed to the article and approved the submitted version.
